# Adhesive Materials Inspired by Barnacle Underwater Adhesion: Biological Principles and Biomimetic Designs

**DOI:** 10.3389/fbioe.2022.870445

**Published:** 2022-04-25

**Authors:** Kesheng Gan, Chao Liang, Xiangyun Bi, Jizhe Wu, Zonghuang Ye, Wenjian Wu, Biru Hu

**Affiliations:** ^1^ College of Liberal Arts and Sciences, National University of Defense Technology, Changsha, China; ^2^ Department of Neurology, Xiangya Hospital, Central South University, Changsha, China

**Keywords:** wet adhesion, barnacle cement proteins, amyloid fibers, spatiotemporally regulated adhesion process, self-assembling peptides, biomimetic adhesive materials

## Abstract

Wet adhesion technology has potential applications in various fields, especially in the biomedical field, yet it has not been completely mastered by humans. Many aquatic organisms (e.g., mussels, sandcastle worms, and barnacles) have evolved into wet adhesion specialists with excellent underwater adhesion abilities, and mimicking their adhesion principles to engineer artificial adhesive materials offers an important avenue to address the wet adhesion issue. The crustacean barnacle secretes a proteinaceous adhesive called barnacle cement, with which they firmly attach their bodies to almost any substrate underwater. Owing to the unique chemical composition, structural property, and adhesion mechanism, barnacle cement has attracted widespread research interest as a novel model for designing biomimetic adhesive materials, with significant progress being made. To further boost the development of barnacle cement–inspired adhesive materials (BCIAMs), it is necessary to systematically summarize their design strategies and research advances. However, no relevant reviews have been published yet. In this context, we presented a systematic review for the first time. First, we introduced the underwater adhesion principles of natural barnacle cement, which lay the basis for the design of BCIAMs. Subsequently, we classified the BCIAMs into three major categories according to the different design strategies and summarized their research advances in great detail. Finally, we discussed the research challenge and future trends of this field. We believe that this review can not only improve our understanding of the molecular mechanism of barnacle underwater adhesion but also accelerate the development of barnacle-inspired wet adhesion technology.

## 1 Introduction

Wet adhesion technology can achieve effective bonding between the interfaces of similar and dissimilar materials in wet or underwater conditions (Cai et al., 2021; Cui and Liu, 2021; Fan and Gong, 2021; Narayanan et al., 2021). It is highly desirable in many different fields, especially in the biomedical field, such as surface modification, wound closure, and hemostatic sealing. Although conventional chemical adhesive materials show strong adhesion ability in the air, their adhesive performance is greatly diminished or even eliminated upon being immersed in water. Intriguingly, several aquatic organisms inhabiting the marine intertidal zones, including mussels (Waite, 2017), sandcastle worms (Stewart et al., 2011), and barnacles (Khandeparker and Anil, 2007; Kamino, 2008; Power et al., 2010; Liang et al., 2019), have successfully evolved into wet adhesion specialists. They can achieve rapid and robust attachment to almost all materials underwater through synthesizing, secreting, and curing biological adhesives. Consequently, learning from the adhesion principles of bioadhesives to engineer artificial adhesive materials provides a promising avenue to address the issue of wet adhesion.

Different biological prototypes have their characteristics and therefore offer rich inspiration for engineering biomimetic adhesive materials (Kamino, 2010b, 2013; Almeida et al., 2020). Mussels mainly rely on foot proteins with abundant 3,4-dihydroxyphenylalanine (Dopa) to ensure underwater attachment. Dopa is a non-natural amino acid that is posttranslationally modified from tyrosine under the catalysis of phenoloxidase *in vivo*. It can not only achieve interfacial adhesion and bulk cohesion through various covalent and non-covalent intermolecular interactions but also cooperate with other neighboring amino acids in the foot proteins to synergistically enhance adhesion (Waite, 2017; Li and Cao, 2019; Saiz-Poseu et al., 2019). To date, by taking advantage of Dopa, chemistry and biochemistry researchers have created various types of mussel-inspired adhesive materials, such as coatings (Lee et al., 2007; Ryu et al., 2018), adhesives (Lee et al., 2011; Yang et al., 2014; Zhang K. et al., 2017; Hauf et al., 2017; Guo et al., 2020), and hydrogels (Zhang C. et al., 2020; Zhang W. et al., 2020). Doubtless, the success of mussel-inspired adhesive materials ranks among the most prominent achievements in biomimetics in the last decade. To construct tubular shelters, sandcastle worms secrete bioadhesives composed of multiple cement proteins to quickly glue sand grains together in seawater. Apart from Dopa residues, these cement proteins also show significantly different net charges under physiological conditions. Leveraging the electrostatic attraction between oppositely charged proteins, the sandcastle worm adhesive spontaneously undergoes a liquid–liquid phase separation (LLPS) process, resulting in a condensed phase designated as coacervate (Zhao et al., 2005). Since adhesives in the form of coacervate have several advantages, including high concentration, low surface energy, anti-dispersing ability, and ease of fabrication (Jho et al., 2017), a large number of biomimetic adhesives have been prepared by physically blending oppositely charged polyelectrolytes (Lim et al., 2010; Shao and Stewart, 2010; Xu et al., 2017).

Barnacles are notorious marine fouling organisms that secrete multi-protein barnacle cement for robust and permanent attachment to almost any kind of underwater substrates (Holm, 2012; Demirel et al., 2017). Distinct from mussel foot proteins and sandcastle worm adhesive, barnacle cement exhibits novel adhesion mechanisms and thus provides another ideal model for the design of biomimetic adhesive materials (Kamino, 2010b; 2013). Moreover, the BCIAMs can be optimally designed by integrating the advantages of mussel- and sandcastle worm–inspired adhesive materials while avoiding their drawbacks. As a consequence, in recent years, an increasing number of studies have been carried out to engineer BCIAMs. To date, numerous prominent results in this field have been achieved. Unfortunately, no relevant reviews summarizing the design strategies and research advances of BCIAMs have been published yet, to the best of our knowledge. In this context, we presented a systematic review of the design concepts and research progress of adhesive materials inspired by barnacle underwater adhesion. At first, the adhesion principles of barnacle cement, which are the source of inspiration for BCIAMs, were briefly introduced. Subsequently, the BCIAMs were reasonably classified into three major categories according to their different design concepts, including biomimetic surface anchors based on natural barnacle cement, recombinant barnacle cement proteins (BCPs) and barnacle-inspired peptides, and biomimetic adhesive materials inspired by the adhesion principles of barnacle cement. Their research progress was summarized in great detail. Finally, the research challenges and future trends of BCIAMs were discussed.

## 2 Adhesion Principles of Barnacle Cement

Understanding the adhesion principles of barnacle cement is a prerequisite of engineering BCIAMs, and a few recent reviews have comprehensively summarized the underwater adhesion mechanism of barnacle cement (Zhang X. K. et al., 2017; Liang et al., 2019). As such, we only provide a brief overview on this topic from three critical facets.

### 2.1 Composition and Microstructure of Barnacle Cement

**FIGURE 1:**
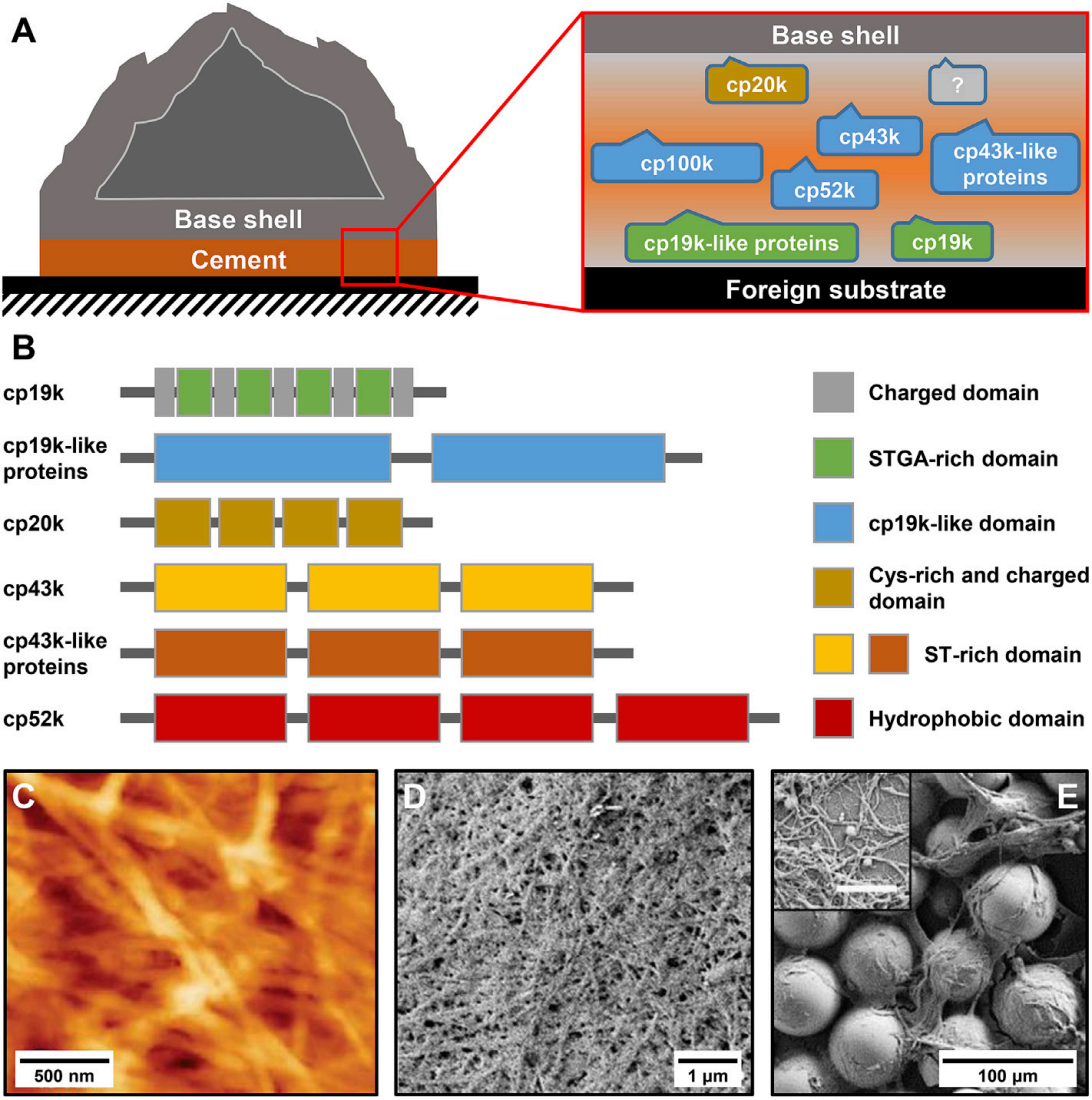
Protein composition and microscale structure of barnacle cement. **(A)** Schematic diagram of an adhered acorn barnacle and the protein composition of its cement. **(B)** Sequence characteristics of different BCPs. **(C)** Atomic force microscope (AFM) image of *Amphibalanus amphitrite* cement. Scanning electron microscope (SEM) image of *A. amphitrite* cement attached to **(D)** silicone panels and **(E)** glass beads. The inset in **(E)** shows magnified cement nanofibers. The scale bar is 500 nm. **(C)** Adapted with permission from Barlow et al. (2010). Copyright 2010, American Chemical Society. **(D,E)** Adapted from So et al. (2016).

Barnacle cement is a multi-protein complex that is synthesized in the cement glands and delivered *via* a series of canals to the narrow gap between the barnacle base shell and the foreign substrate, where it gradually assembles and crosslinks to achieve robust underwater adhesion (Figure 1A **)**. In the past two decades, Kamino et al. (2000); Kamino (2001); Urushida et al. (2007); Kamino et al. (2012) identified several cement proteins from *Megabalanus rosa* and successfully cloned the gene sequences of four cement proteins, including cp100k, cp52k, cp20k, and cp19k (they were named after their apparent molecular weights). Employing homologous amplification technology, they also isolated the gene sequences of *Balanus albicostatus* cp19k and cp20k and *Balanus improvisus* cp19k (Mori et al., 2007; Urushida et al., 2007). Followed by these pioneering studies, researchers have continued to obtain additional homologous genes of BCPs through gene cloning and RNA sequencing from a variety of barnacle species, including *Amphibalanus amphitrite* (He et al., 2013; Wang et al., 2015; He L.-S. et al., 2018), *Tetraclita japonica formosana* (Lin et al., 2014), *Pollicipes pollicipes* (Rocha et al., 2019), and *Megabalanus volcano* (Yan et al., 2020). In addition, So et al. (2016) identified a 43-kDa cement protein (cp43k) from *A. amphitrite* and obtained its gene sequence. In particular, the authors also discovered many cp43k- and cp19k-like proteins, which show a similar amino acid composition to cp43k and cp19k but have varied molecular weights. In this view, the cp68k identified by Kamino (2006) may be a cp43k-like protein for their similar amino acid compositions. Several latest omics investigations further confirmed that these different types of BCPs are widespread in different barnacle species (Domínguez-Pérez et al., 2021; Lin et al., 2021; Schultzhaus et al., 2021). Notably, apart from these major BCPs, there remains a trace amount of other components such as enzymes and small molecules in barnacle cement, which have not been fully characterized yet.

The amino acid compositions of different BCPs have been intensively analyzed (Kamino, 2008; Liang et al., 2019). Briefly, cp100k and cp52k have a high percentage of hydrophobic amino acids, cp20k is dominated by Cys and charged amino acids, and cp43k and cp19k show strong bias to six amino acids (Ser, Thr, Gly, Ala, Val, and Lys). Despite their remarkably different amino acid compositions, all BCPs identified thus far contain no Dopa residues, indicating that barnacle cement evolves a Dopa-independent underwater adhesion strategy that relies on the unique combination and arrangement of natural amino acids. Additionally, almost all characterized BCPs show specific patterned sequence features (Figure 1B), which are rarely observed in mussel foot proteins and sandcastle worm cement proteins. Research has indicated that cp19k resembles a block copolymer composed of blocks that are rich in charged amino acids (charged domains) and blocks that are dominated by Ser (S), Thr (T), Gly (G), and Ala (A) (STGA-rich domains) (Kamino, 2008; Liang et al., 2019), while the cp19k-like proteins contain several tandem cp19k-like domains (So et al., 2016). Similarly, cp20k consists of multiple non-strictly repeated fragments with abundant Cys and charged amino acids (Cys-rich and charged domains) (Kamino, 2001). Both cp43k and cp43k-like proteins contain three tandem domains with abundant Ser and Thr (ST-rich domains) (So et al., 2016). The primary structure of cp52k consists of four repetitive hydrophobic domains (Kamino et al., 2012), whereas the hydrophobic cp100k has no obvious sequence repeats. By analyzing the conformation of bacterial recombinant BCPs, Wang et al. (2018) and Liang et al. (2018) consistently found that the secondary structure of cp19k is dominated by random coils and β-sheets. Mohanram et al. (2019) reported the tertiary structure of cp20k and discovered that this protein contains three major folded domains, and each of them has several β-strands. The conformation of other BCPs has not been investigated yet primarily because their heterogeneous expression is full of challenges.

The adhesion ability of adhesives is not only determined by their chemical compositions but also related to their microstructures. To this end, several studies have examined the microscale morphologies of barnacle cement. By using atomic force microscopy (AFM) to observe *A. amphitrite* cement, Barlow et al. (2010) found that it consists of dense and interwoven nanofibers (Figure 1C). These nanofibers were thought to be amyloid fibers since they contained abundant β-sheet secondary structures and could bind thioflavin T, an amyloid structure-specific fluorescent dye. In another study, So et al. (2016) carefully collected the cement of *A. amphitrite* attached to silicone panels and glass beads and examined their microstructures using scanning electron microscopy (SEM). Consistently, they observed dense fibrous structures on both substrates (Figures 1D,E **)**. Zheden et al. (2015) collected a special foamy cement from the gooseneck barnacle *Dosima fascicularis*. The macroscale morphology and mechanical property of this cement are significantly different from those of acorn barnacle cement. Nevertheless, microscale morphological observations confirmed that it also contains massive nanofibers. Overall, although different barnacle cement may show obvious differences in macroscopic morphologies, their microstructures are all composed of nanofibers. The nanofiber structures of barnacle cement are significantly different from the sponge-like morphologies of the mussel adhesive plaque and the porous solid–like structures of the sandcastle worm holdfast (Stevens et al., 2007; Waite, 2017), suggesting that BCPs possess the unique ability to self-assemble into nanofibers.

### 2.2 Surface Adhesion and Bulk Cohesion Mechanisms of Barnacle Cement

To successfully bond similar and dissimilar materials together, multi-protein bioadhesives must not only couple with diverse foreign substrates (surface adhesion) but also polymerize themselves (bulk cohesion). Accordingly, revealing how barnacle cement fulfills surface adhesion and bulk cohesion is key to elucidating the mechanism of barnacle underwater adhesion. As of now, several hypothesized models of barnacle cement adhesion and cohesion mechanisms have been proposed.

Based on the structural and functional characterizations of different BCPs, Kamino (2006) first proposed a molecular model of barnacle cement underwater adhesion and updated it around 10 years later (Kamino, 2016). In the latest model, he argues that cp19k, cp20k, and cp68k (a putative cp43k-like protein) are interface cement proteins, which are located at the exterior of barnacle cement and bind non-covalently to the barnacle base shell and foreign substrates. In contrast, cp52k and cp100k are bulk cement proteins, which are distributed in the interior of barnacle cement and polymerize *via* conformation optimized intermolecular non-covalent interactions. The interface and bulk cement proteins further interplay to achieve successful underwater adhesion. As no Dopa residues, intermolecular disulfide bonds, and enzyme-catalyzed covalent crosslinks were detected in barnacle cement, the model insisted that different BCPs use sequence and conformation optimized non-covalent interactions to collaboratively achieve underwater adhesion. Currently, the model is widely accepted; however, its foundation, the spatial distribution of BCPs, needs to be substantiated.

By collecting and analyzing natural barnacle cement (which refers to the unsolidified liquid barnacle cement stored in the glands), Dickinson et al. (2009) detected transglutaminase, an important enzyme involved in the blood coagulation process. Moreover, they discovered that polymerized barnacle cement shows nanoscale fibrous networks resembling the microscale morphology of blood clots. On the basis of these findings, they speculated that barnacle cement polymerization and blood coagulation might share analogous molecular mechanisms. This speculation is supported by the evolutionary concept that many biochemical mechanisms essential to survival are highly conserved. However, it is still in debate primarily because the collected liquid barnacle cement might be contaminated by body liquid and the reaction products have not been detected yet (Kamino, 2010a). Moreover, this hypothesis did not shed light on how barnacle cement achieves surface adhesion.

In addition, So et al. (2017) proposed an enzyme-catalyzed barnacle cement polymerization model based on the discovery that barnacle cement contains different enzymes such as peroxidase and lysyl oxidase. In the model, the authors argue that BCPs first self-assemble into amyloid-like fibers through non-covalent interactions. Forming amyloid-like fibers can greatly contribute to wet adhesion. First, it can enhance the cohesion of adhesive proteins because the abundant sacrificial bonds in amyloid-like fibers can effectively dissipate energy applied to them (Fukuma et al., 2006). Second, amyloid-like fibers can exhibit strengthened surface adhesion owing to the bending energy penalty (Zhang Y. et al., 2017) as well as the unique sequence properties and structural features of their subunits (DeBenedictis et al., 2016). Moreover, the different functional groups present on the surface of amyloid-like structures can form multiple adhesive interactions with the substrate to promote adhesion (Gu et al., 2018). Subsequently, these amyloid-like fibers are covalently crosslinked to further enhance cohesion under the catalysis of different enzymes. Specifically, peroxidase catalyzes the conversion of free catechol small molecules in barnacle cement into quinones, which further react with amine groups in BCPs to form stable quinone protein crosslinking, while lysyl oxidase directly catalyzes multivalent crosslinks of lysine in BCPs. However, the hypothesis needs further confirmation since the crosslinked products have not been detected in polymerized barnacle cement yet.

### 2.3 Spatiotemporal Regulation of Barnacle Underwater Adhesion

Apart from the unique chemical compositions and microscale structures as well as adhesion and cohesion mechanisms, the spatiotemporal regulation of the adhesion process of bioadhesives is also indispensable for successful underwater adhesion. Several common regulating strategies adopted by different organisms have been revealed, which include the controllable synthesis and processing of bioadhesives (Power et al., 2010; Gohad et al., 2014; Priemel et al., 2017; Fears et al., 2018), pretreatment of foreign substrates (Gohad et al., 2014; Waite, 2017; Fears et al., 2018), and regulating the secretion sequence of different components (Gohad et al., 2014; Petrone et al., 2015; He Y. et al., 2018; Fears et al., 2018).

In general, the glands in which bioadhesives are synthesized and the seawater in which bioadhesives function show distinct environmental factors such as pH and ionic strength, and many marine organisms take advantage of this to regulate adhesion. For example, the gland environment of barnacles is acidic and has low-ionic strength, while the seawater is alkaline and has high-ionic strength (Power et al., 2010). Through *in vitro* experiments, our group found that cp19k can self-assemble into short nanofibers in a simulated gland environment (Liang et al., 2018) and longer amyloid-like fibers in artificial seawater at a faster self-assembling rate (Liu et al., 2017), suggesting that barnacles may release pre-assembled fibrous cement for underwater adhesion. The possible advantages include preventing barnacle cement from being severely diluted by seawater and serving as seeds to induce and accelerate self-assembly. Likewise, when mussels deposit foot proteins, they first create a closed acidic niche to keep Dopa in its reduced form to ensure robust interfacial adhesion. Subsequently, when mussel foot proteins contact the alkaline seawater, Dopa undergoes oxidative crosslinking to enhance cohesion (Waite, 2017). In addition, under physiological conditions, sandcastle worm cement spontaneously forms special coacervate through an LLPS process. Upon secreted into seawater, the coacervate quickly turns into insoluble sediments and firmly adheres to the substrate since the changing environment breaks its equilibrium state (Wang and Stewart, 2013).

Preparing the adherents to keep them clean and dry is critical to biological underwater adhesion, which is mainly fulfilled by regulating the secretion sequence of different components of bioadhesives. Gohad et al. (2014) found that the permanent adhesive secreted by barnacle larvae is a biphasic system consisting of lipids and phosphoproteins. Under fine biological control, the larvae first release hydrophobic lipids to remove surface-bound water layers to create a conducive local environment and then secrete phosphoproteins for adhesion. In a subsequent study, Fears et al. (2018) found that adult barnacles also release a fluid containing lipids and reactive oxidative species ahead of depositing proteinaceous cement. This fluid probably plays a role in removing organic matters and hydration layers on the surface in order to ensure effective adhesion. Similarly, He Y. et al. (2018) discovered that mussels also secrete hydrophobic lipids to repel interfacial water layers before depositing foot proteins. Furthermore, the secretion sequence of different mussel foot proteins is also strictly controlled (Petrone et al., 2015). In summary, regulating the secretion sequence of different components to create a hydrophobic local environment represents a common strategy of different marine organisms to achieve underwater adhesion.

## 3 Barnacle Cement–Inspired Adhesive Materials

Based on the understanding of the adhesion principles of barnacle cement, researchers have engineered many different BCIAMs so far. These BCIAMs can be reasonably classified into three major categories based on their different design strategies. The first is biomimetic surface anchors based on natural barnacle cement, the second is recombinant BCPs and barnacle-inspired peptides, and the third is biomimetic adhesive materials designed from the adhesive principles of barnacle cement. In this section, we will systematically summarize the research advances of different categories of BCIAMs.

### 3.1 Biomimetic Surface Anchors Based on Natural Barnacle Cement

As a bioadhesive, barnacle cement can non-specifically adhere to various surfaces and form a stable adsorption layer even in harsh environments. Due to this property, collected natural barnacle cement, namely, the unsolidified liquid barnacle cement stored in the glands, can be readily used for the coating of different types of materials. Moreover, the abundant active groups (e.g., –OH, –NH_2_, and–SH) in barnacle cement allow further conjugation of different functional polymers to tailor surfaces with specific functionalities, that is, natural barnacle cement can be applied as biomimetic surface anchors.

**FIGURE 2:**
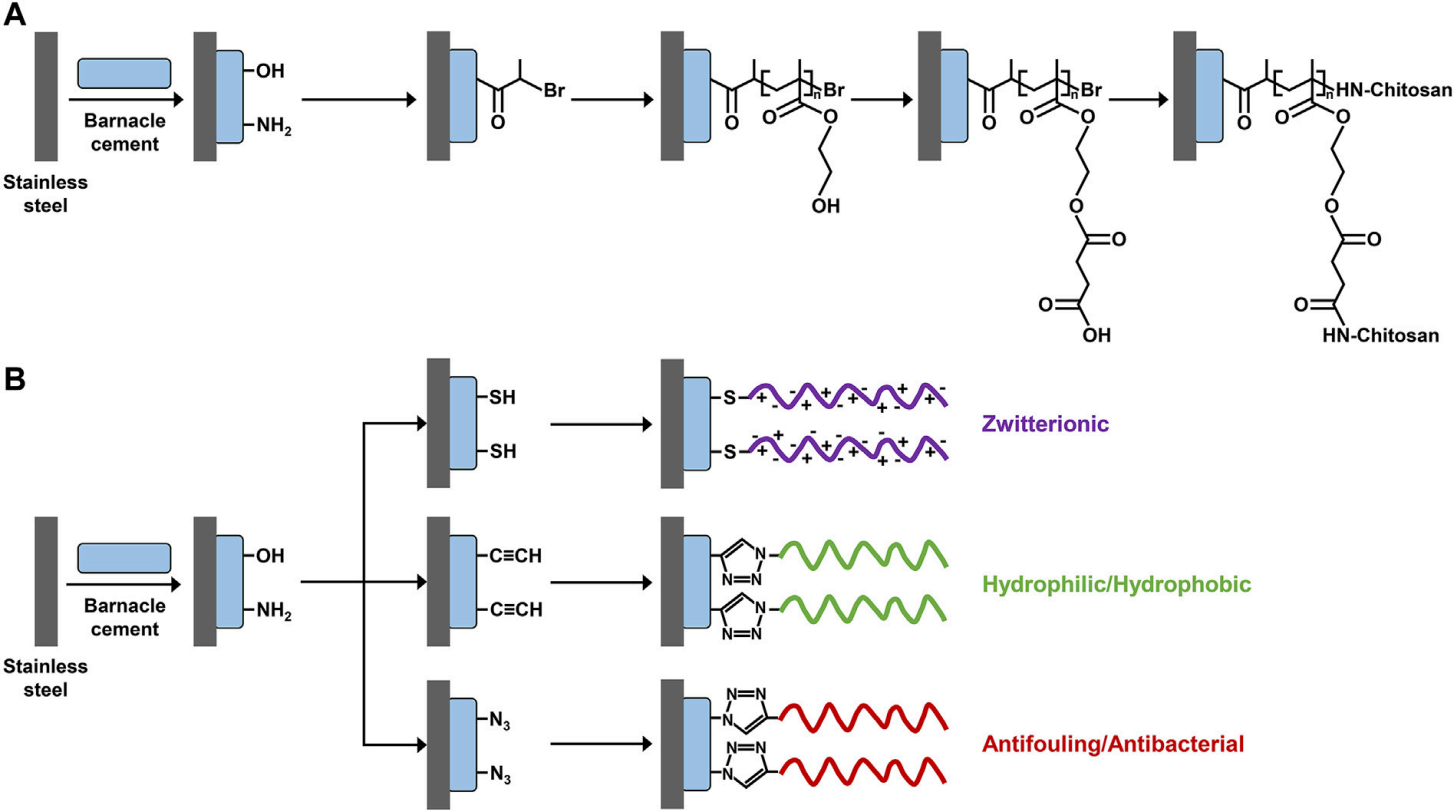
Scheme of surface modification using barnacle cement as a biomimetic anchor. Collected fresh liquid barnacle cement is first coated on a stainless steel panel to introduce abundant hydroxyl and amine groups. Then, different functional polymer brush structures are constructed on the stainless steel panel through **(A)** surface-initiated atom transfer radical polymerization and **(B)** click chemistry. **(A)** Adapted with permission from Yang et al. (2011). Copyright 2011, American Chemical Society. **(B)** Adapted with permission from Yang et al. (2013). Copyright 2013, American Chemical Society.

The concept of using liquid barnacle cement as biomimetic surface anchors for surface functionalization was first proposed and demonstrated by Yang et al. (2011). As illustrated in Figure 2A, they first uniformly coated a stainless steel panel with freshly collected liquid barnacle cement and then grafted chitosan to the barnacle cement adhesive layer *via* a series of chemical reactions including atom transfer radical polymerization, esterification, and amidation, by taking advantage of the high abundance of hydroxyl and amine groups of barnacle cement. In this way, a stable hydrophilic polymer brush structure was successfully prepared on the stainless steel panel, endowing it with the dual functions of anti-protein adhesion and bactericide. In comparison, the grafting efficiency of chitosan when using barnacle cement as surface anchors was comparable to that when using polydopamine, a famous mussel-inspired adhesive material. In a subsequent study, the same group further expanded the design (Yang et al., 2013). They first introduced thiol, alkyl, or azido groups to the barnacle cement pre-coated stainless steel panel. Then, polymers with different structural and functional properties were specifically conjugated to this surface *via* click chemistry (Figure 2B). The constructed polymer brush structures on the stainless steel panel *via* this approach showed strong stability and could maintain their integrity after being soaked in phosphate-buffered saline (PBS) for 1 month.

Making use of natural barnacle cement as biomimetic surface anchors for surface modification has many advantages, including good stability, substrate-independent adhesion, rich functional groups, biocompatibility, and environmental friendliness (Pranantyo et al., 2017). However, it is quite challenging to collect a large quantity of liquid barnacle cement for large-scale applications, primarily because barnacle cement has low production and can be easily contaminated during sampling. As a result, only a few studies have attempted to directly use natural barnacle cement to design BCIAMs to the best of our knowledge.

### 3.2 Recombinant BCPs and Barnacle-Inspired Peptides

As discussed previously, the direct use of natural barnacle cement is greatly limited by its low yield. To overcome the issue, many researchers have attempted to artificially reconstitute simplified versions of barnacle cement in a reductionist way by synthesizing key recombinant BCPs or barnacle-inspired peptides that retain the adhesion ability and biocompatibility of natural barnacle cement while can be produced on a large scale (Stewart, 2011). These recombinant BCPs and barnacle-inspired peptides constitute the second group of BCIAMs.

#### 3.2.1 Recombinant BCPs

Urushida et al. (2007) first expressed recombinant *Megabalanus rosa* cp19k (rMrcp19k) in *Escherichia coli*. Employing surface plasmon resonance, the authors discovered that rMrcp19k shows strong adsorption ranging from tens to hundreds of nanograms per centimeter square (ng/cm^2^) on different substrates. Accordingly, they inferred that cp19k is capable of non-specifically coupling with various surfaces and that it is a key adhesive protein in barnacle cement. Wang et al. (2018) expressed rMrcp19k and recombinant *Balanus albicostatus* cp19k (rBalcp19k) and quantitatively compared their adsorption on silica by spectroscopic ellipsometry. They found that the former has approximately one-half the adsorption of the latter, suggesting that rBalcp19k may be more promising for developing protein-based adhesive materials. Soon after, our group prepared high-purity rBalcp19k and examined its microscale adhesion strength by AFM-based colloidal probe force spectroscopy (Liang et al., 2018). We found that rBalcp19k shows comparable adhesion strength to Cell-Tak, a commercial cell and tissue adhesive, under basic and weakly acidic conditions. Moreover, we noticed that the adhesion property of rBalcp19k is enhanced after it self-assembles into nanofibers. Producing other bacterial recombinant BCPs is more challenging since they either have extremely abundant Cys or numerous hydrophobic amino acids. Mori et al. (2007) successfully expressed recombinant *Megabalanus rosa* cp20k (rMrcp20k) in *E. coli*. Its adsorption ability was indirectly measured by quantifying soluble rMrcp20k after immersing different particles in rMrcp20k solution for some time. Through this way, the authors found that rMrcp20k exhibits relatively high adsorption (in the range of tens to hundreds of ng/cm^2^) on calcite, metals, and metal oxides but low adsorption on glass and polystyrene. More recently, by using a quartz crystal microbalance, Murugan et al. (2020) discovered that rMrcp20k shows strikingly high adsorption on AH36 steel sheets (approximately 500 ng/cm^2^), much higher than that of bovine serum albumin and lysozyme. These results indicate that cp20k can efficiently bind to certain types of materials. In addition, So et al. (2016) expressed recombinant *A. amphitrite* cp43k and Zeng (2016) produced recombinant *M. rosa* cp52k. However, neither study examined the adhesion ability of recombinant BCPs.

**FIGURE 3:**
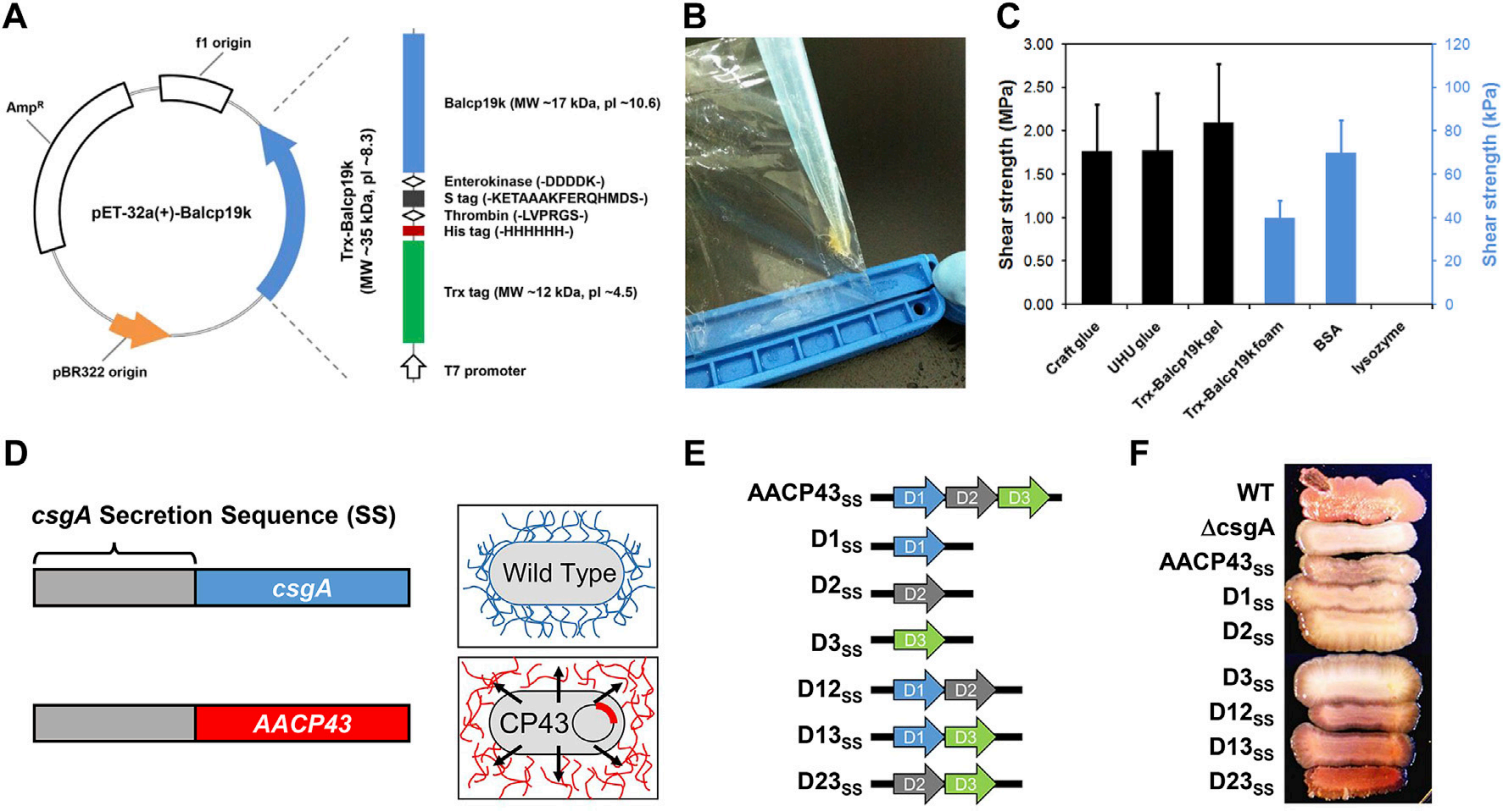
Expression and characterization of different recombinant BCPs. **(A)** Structural illustration of Trx-Balcp19k. **(B)** Photograph of the tacky condensates (Trx-Balcp19k gel) formed by Trx-Balcp19k after being dialyzed against pure water. **(C)** Adhesive shear strength of different samples. Craft glue and UHU glue are commercially available chemical adhesives, and the Trx-Balcp19k foam represents unaggregated Trx-Balcp19k. **(D)** Scheme showing the design concept of AACP43_SS_. **(E)** Structural illustrations of AACP43_SS_ and its mutants. **(F)** Congo red staining results of wild-type (WT) bacterial strains, ΔcsgA, and ΔcsgA expressing AACP43_SS_ and its mutants. **(A–C)** Adapted from Liang et al. (2015). **(D–F)** Adapted with permission from Estrella et al. (2021). Copyright 2021, American Chemical Society.

Taken together, cp19k can non-selectively bind to various substrates and be more easily expressed in *E. coli* than other BCPs. Therefore, cp19k provides an ideal candidate for developing protein-based adhesive materials. However, to engineer cp19k-based adhesive materials, it is essential to further improve their adhesion ability and production. As shown in Figure 3A, our group previously constructed a fusion protein termed Trx-Balcp19k, which contains a large thioredoxin tag (Trx) at the N-terminus and *Balanus albicostatus* cp19k (Balcp19k) at the C-terminus (Liang et al., 2015). Owing to the cooperative roles between Trx and Balcp19k, the fusion protein not only exhibited a high expression level in *E. coli* but also spontaneously aggregated into a super tacky condensate (abbreviated as Trx-Balcp19k gel) on the dialysis membrane during dialysis against pure water (Figure 3B). The Trx-Balcp19k gel showed a high adhesive shear strength of 2.10 ± 0.67 MPa on aluminum adherents in air, comparable to that of several commercial adhesives (Figure 3C **)**. This cp19k-based adhesive material is promising for its convenient fabrication, strong adhesion, and good biocompatibility. However, its wet adhesion ability has not yet been determined. Similarly, Tilbury et al. (2019) linked a bacterial molecular chaperone called trigger factor (TF) to the N-terminus of *Pollicipes pollicipes* cp19k (Ppolcp19k) and created a hybrid protein named TF-rPpolcp19k-his, where “his” stands for the hexahistidine tag. As expected, TF-rPpolcp19k-his showed an increased expression level in *E. coli*. Furthermore, surface coat analysis indicated that it has higher adsorption on both hydrophilic and hydrophobic surfaces than TF tag-free rPpolcp19k-his. From the aforementioned two examples, it can be seen that rationally combining BCPs with other protein tags can significantly enhance the yield and adhesion ability of target proteins. This provides a feasible approach for developing barnacle cement–inspired protein-based adhesive materials and deserves further study.

In a recent study, Estrella et al. (2021) designed a recombinant protein denoted AACP43_SS_, which integrates the secretion sequence (SS) of bacterial curli protein csgA and *Amphibalanus amphitrite* cp43k (AACP43) **(** Figure 3D). AACP43_SS_ was successfully expressed in a csgA single-gene knockout *E. coli* strain (ΔcsgA) and secreted to the extracellular environment under the aid of co-expressed csgG, where it further self-assembled into Congo red–sensitive typical amyloid fibers. In contrast, ΔcsgA bearing no *AACP43*_*SS*_ gene failed to be stained by Congo red because of no self-assembled amyloid fibers. Considering that AACP43 contains three tandem conserved domains, the authors also constructed a series of mutants by deleting one or two domains (Figure 3E). These mutants were expressed in the same way to identify the key domains for self-assembly. It was found that all mutants retain the self-assembling property to form amyloid fibers with only slight differences in the degree of self-assembly as indicated by the different intensities of Congo red staining (Figure 3F). Interestingly, the one deleting the first domain (D23_SS_) showed the strongest self-assembling ability and outperformed native AACP43. Altogether, this study subtly confirmed the self-assembling ability of cp43k and revealed the relationship between its self-assembling property and structure. However, it did not check the adhesion ability of cp43k and its mutants. Genetically editing bacteria to produce functional extracellular substances and using them together with live bacteria to fabricate engineered live materials is a research focus in the field of synthetic biology (Nguyen et al., 2018; Gilbert and Ellis, 2019; Rodrigo-Navarro et al., 2021; Tang et al., 2021). In this way, researchers have developed many engineered live adhesive materials (Zhang et al., 2019; An et al., 2020), which have several advantages such as no need for purification of target proteins, self-repairing, programmability, and so on. Therefore, the aforementioned expression platform for producing recombinant BCPs established by Estrella et al. (2021) can also be harnessed to engineer new living adhesive materials.

#### 3.2.2 Barnacle-Inspired Peptides

It is well known that peptides have multiple advantages compared with proteins, including smaller molecular weight, lower structural complexity, easier chemical synthesis, and so on. Therefore, designing and screening barnacle-inspired peptides and using them instead of intact BCPs as building blocks to fabricate biomimetic functional materials are becoming increasingly focused. Indeed, based on the structural and functional properties of different BCPs, many barnacle-inspired peptides have been designed so far (Table 1).

**TABLE 1:** Summary of barnacle-inspired peptides.

Protein	Peptide	Sequence^a^	Assembly	Reference
Mrcp20k	cMr20-S5	SKLPCNDEHPCYRKEGGVVSCDCK	Membrane consists of nanofilaments and mesh-like structures composed of nanofilament bundles	Nakano et al. (2007)
	cMr20-S5 (Ser)	SKLPSNDEHPSYRKEGGVVSSDSK	Membrane consists of nanofilaments	
	cMr20-S6	KTITCNEDHPCYHSYEEDGVTKSDCDCE	Nanofilaments	
Mrcp52k	R1-3 (R-Y)	RRKYSGILGDLIQVAVIRYY	Nanofibrils	Nakano and Kamino, (2015)
	R1-5	YNAKYVTGFIRGFMGYM	N.A	
	R2-3	RSRYSGVQLDLLCLAALRYY	N.A	
	R2-4	FDYALEHSLKSTA	N.A	
	R3-3	KKKYSGYRSDLLQLAAIRYCLY	N.A	
	R4-1	GLPKLSMPQYSLSGLMSYI	N.A	
	R4-3	KSRYSGIQADLLQLCAIRYY	N.A	
	R4-4	FGTIFQQYLGSQN	N.A	
	RGDS-R-Y	RGDSGRRKYSGILGDLIQVAVIRYY	Nanofiber network	Fujii et al. (2021)
	R-RGD-Y	RRKYSGIRGDLIQVAVIRYY	Nanofiber network	
	R-Y-RGDS	RRKYSGILGDLIQVAVIRYYGRGDS	Nanofiber network	
cp19k-like proteins	BCP1C	QTGYTRGGAAVSSTGATQGAGSLDLAIDGPGGFKARSK	Long nanofibrils	So et al. (2019)
	BCP2	AVGNSGVSGSGVSIGDSGFRQKTQT	Short nanofibrils	
	BCP2C	AVGNSGVSGSGVSIGDSGFRQKTQTNSEAGSKGTKRA	Long nanofibrils	
	mutBCP1	QTGYTRGGAAVSSTGATQCAGS	Short nanofibers	
cp19k	Bp1	GSGSVPPPCD	N.A	Raman et al. (2017)
	Bp2	GSKLDLLTDG	N.A	

a The termini of some peptides have been modified.

**FIGURE 4:**
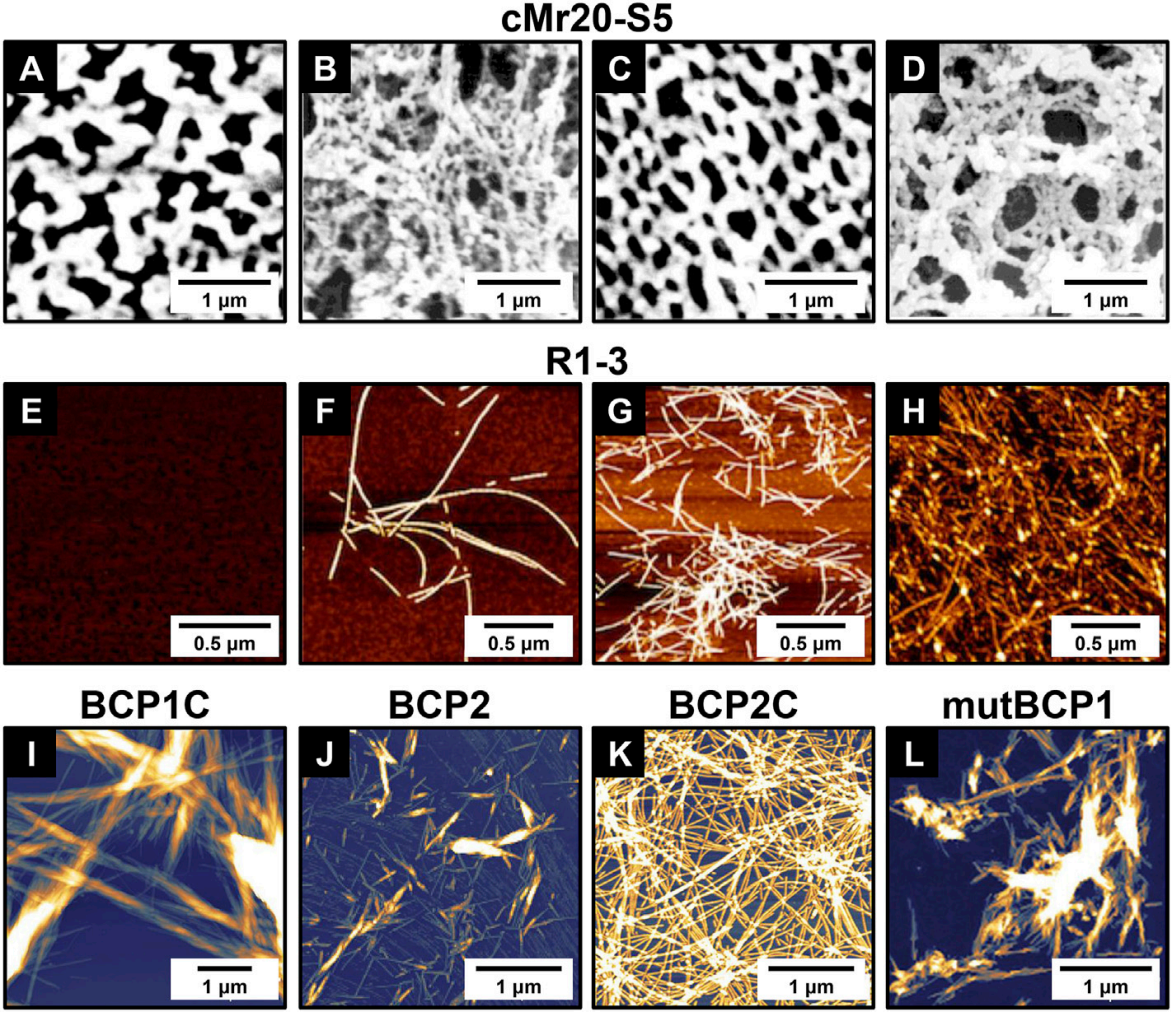
Self-assembled structures of representative barnacle-inspired peptides. **(A)** AFM and **(B)** SEM images of cMr20-S5 assemblies in PBS supplemented with 1 M NaCl; **(C)** AFM and **(D)** SEM images of cMr20-S5 assemblies in 1 M NaCl with alkaline preincubation. AFM images of R1-3 incubated at pH 4.0 for **(E)** 5 min or **(F)** > 2 weeks and at pH 9.0 for **(G)** 5 min or **(H)** > 2 weeks. AFM images showing the self-assembled nanostructures from **(I)** BCP1C, **(J)** BCP2, **(K)** BCP2C, and **(L)** mutBCP1. **(A–D)** Adapted with permission from Nakano et al. (2007). Copyright 2007, American Chemical Society. **(E–H)** Adapted with permission from Nakano and Kamino (2015). Copyright 2015, American Chemical Society. **(I–L)** Adapted with permission from So et al. (2019). Copyright 2019, American Chemical Society.

As illustrated in Figure 1B, the primary structure of cp20k can be divided into multiple non-strict repeats according to the regular alignment of Cys. Inspired by this feature, Nakano et al. (2007) designed two biomimetic peptides from the fifth and sixth repeats of Mrcp20k, which were named cMr20-S5 and cMr20-S6 (Table 1), respectively. In neutral PBS supplemented with 1 M NaCl, cMr20-S5 formed a macroscopically distinguishable membrane composed of interwoven nanofilaments (Figures 4A,B). Under the same conditions, cMr20-S5 (Ser), a Cys substituted mutant of cMr20-S5 (Table 1), and cMr20-S6 self-assembled into similar nanofilaments, but the former formed the macroscopic membrane while the latter failed, suggesting that the self-assembly of cMr20-S5 is independent of intermolecular disulfide bonds. In comparison, after being preincubated in alkaline conditions and supplemented with 1 M NaCl, cMr20-S5 formed intramolecular disulfide bonds and β-sheet structures and self-assembled into a mesh-like structure composed of bundles of nanofilaments (Figures 4C,D). This study reported barnacle-inspired self-assembling peptides for the first time. Recently, Mohanram et al. (2019) revealed the three-dimensional structure of recombinant Mrcp20k by combining multi-dimensional nuclear magnetic resolution and molecular dynamics simulations. They discovered that the β-sheet structure (β7–β8) composed of the seventh and eighth β-strands in the protein is highly stable and may function as a nucleus to induce amyloid self-assembly. Consistently, the self-assembling peptide cMr20-S5 also contains the amino acid sequence corresponding to β7–β8.

Later on, Nakano and Kamino (2015) noticed that the sequence of Mrcp52k consists of four large repeats and each of them can be further divided into five different regions. Based on this, they designed a series of Mrcp52k-derived peptides designated as Rn-m, where n and m denote the numerical order of the repeats and regions, respectively. Thioflavin T staining screened eight peptides that might be able to self-assemble into amyloid fibrils at alkaline or high-ionic strength conditions (Table 1). Further exploring the self-assembly of R1-3, one of the eight potential self-assembling peptides, revealed that under acidic conditions (pH 4.0), it can form amyloid fibrils only after a long period of incubation (Figures 4E,F). On the contrary, under basic conditions, R1-3 undergoes a conformation transition to form an ordered β-sheet structure and quickly self-assembles into amyloid-like fibrils within a few minutes (Figure 4G) and gradually grows into long fibrils after 2 weeks (Figure 4H). As for the other seven Mrcp52k-derived peptides, their self-assembling properties have not been examined. To enhance the biological activity of R1-3 (or R-Y), the cell adhesion motif RGD was incorporated into its different positions (Fujii et al., 2021). It was found that when RGD was added to the N-terminus or center of R-Y, the resulting new peptides RGDS-R-Y or R-RGD-Y (Table 1) can retain their ability to form β-sheet secondary structures and self-assemble to generate hydrogels. Moreover, on substrates coated with these two peptide hydrogels, cell adhesion was greatly promoted. In contrast, when RGD was introduced to the C-terminus of R-Y, the resulting new peptide R-Y-RGDS (Table 1) lost its ability to form β-sheets and hydrogels.

Recently, So et al. (2019) found that the primary structures of the cp19k-like proteins contain multiple cp19k-like domains (Figure 1B), and each domain consists of alternating uncharged low-complexity fragments and charged variable fragments. Inspired by this feature, the authors rationally designed four blocked peptides (BCP1C ∼ BCP4C) and four simple peptides (BCP1∼BCP4). Each blocked peptide contains a low-complexity fragment and a succeeding charged fragment while the simple peptides were designed by simply removing the charged fragment of the corresponding blocked peptides. AFM imaging found that three of the eight biomimetic peptides, including BCP1C, BCP2, and BCP2C, self-assembled into amyloid fibrils in seawater (Figures 4I–K). Although BCP1 did not self-assemble, its mutant mutBCP1 which replaces a Gly of BCP1 with Cys successfully self-assembled into short nanofibers (Figure 4L **)**. The sequence information of the four self-assembling peptides is summarized in Table 1. It is noteworthy that owing to the different sequence patterns, the blocked peptides (BCP1C and BCP2C) and the simple peptide (BCP2) showed different self-assembling pathways and thus formed distinct assemblies. The former self-assembled into long amyloid fibrils resembling those in natural barnacle cement by forming antiparallel β-sheet structures, which can serve as seeds to induce or accelerate the self-assembly of other peptides. In contrast, the latter formed short branching nanofibrils by forming parallel β-sheet structures and did not have the function of self-assembling nuclei.

Kamino (2006) first noted that the sequence of Mrcp19k consists of two types of alternating fragments. One is rich in Ser, Thr, Gly, and Ala (STGA-rich fragment) and the other is dominated by charged and hydrophobic amino acids (e.g., Lys and Val). Unfortunately, the details of the fragments were not reported. Through in-depth sequence alignment and amino acid distribution analysis, Liang et al. (2019) unambiguously identified the different fragments in multiple cp19k homologs. According to this block copolymer-like sequence property of cp19k, our group designed and synthesized a series of cp19k-derived peptides. Currently, their self-assembling abilities are under investigation. In addition, using two distinct and recurring sequences in cp19k as templates, Raman et al. (2017) designed two cp19k-inspired peptides denoted Bp1 and Bp2 (Table 1). Non-interacting sequences GSGS and GS were introduced at the N-terminus of Bp1 and Bp2, respectively, such that they have the same length. In terms of amino acid composition, Bp1 is mostly hydrophobic with a negatively charged tail while Bp2 has a mixture of hydrophobic and charged amino acids. Using surface force apparatus, the authors found that both peptides can effectively bridge mica and hydrophobic surfaces, but show much weaker adhesion between mica and hydrophilic surfaces.

Overall, the vast majority of studies on barnacle-inspired peptides has focused on their self-assembling properties, primarily because self-assembling peptides show potential applications in many areas (Levin et al., 2020). Furthermore, self-assembling into amyloid-like nanofibers is unique to BCPs, which has not been observed in mussel foot proteins and sandcastle worm cement proteins. Consequently, many barnacle-inspired self-assembling peptides have been revealed. In comparison, only Raman et al. (2017) examined the adhesion properties of two cp19k-inspired peptides. In addition, no one has investigated the relationship between the self-assembly and adhesion properties of barnacle-inspired peptides. However, it is critical for the engineering of advanced adhesive materials that combine the self-assembling property and adhesion ability. Therefore, more efforts should be made to address this issue in the future.

### 3.3 Biomimetic Adhesive Materials Designed From the Adhesive Principles of Barnacle Cement

The third category of BCIAMs refers to the adhesive materials that imitate the different adhesive principles of barnacle cement (e.g., the unique amino acid compositions of BCPs, amyloid fibers, and the spatiotemporally regulated adhesion process). Their prominent characteristic is that they are all fabricated with non-barnacle cement-originated substances. According to the distinct adhesive principles, these BCIAMs can be further classified into different subcategories. In the next, their research progress will be separately discussed.

#### 3.3.1 BCIAMs Mimicking the Unique Amino Acid Compositions of BCPs

The amino acid compositions of BCPs are largely different from those of mussel foot proteins and sandcastle worm cement proteins. In particular, BCPs do not have any posttranslationally modified amino acids (e.g., Dopa and phosphorylated Ser) while both mussel foot proteins and sandcastle worm cement proteins contain high percentages of these amino acids. Thus, the unique compositions of common amino acids in BCPs contribute a lot to their wet adhesion and provide important inspirations for the design of BCIAMs.

**FIGURE 5:**
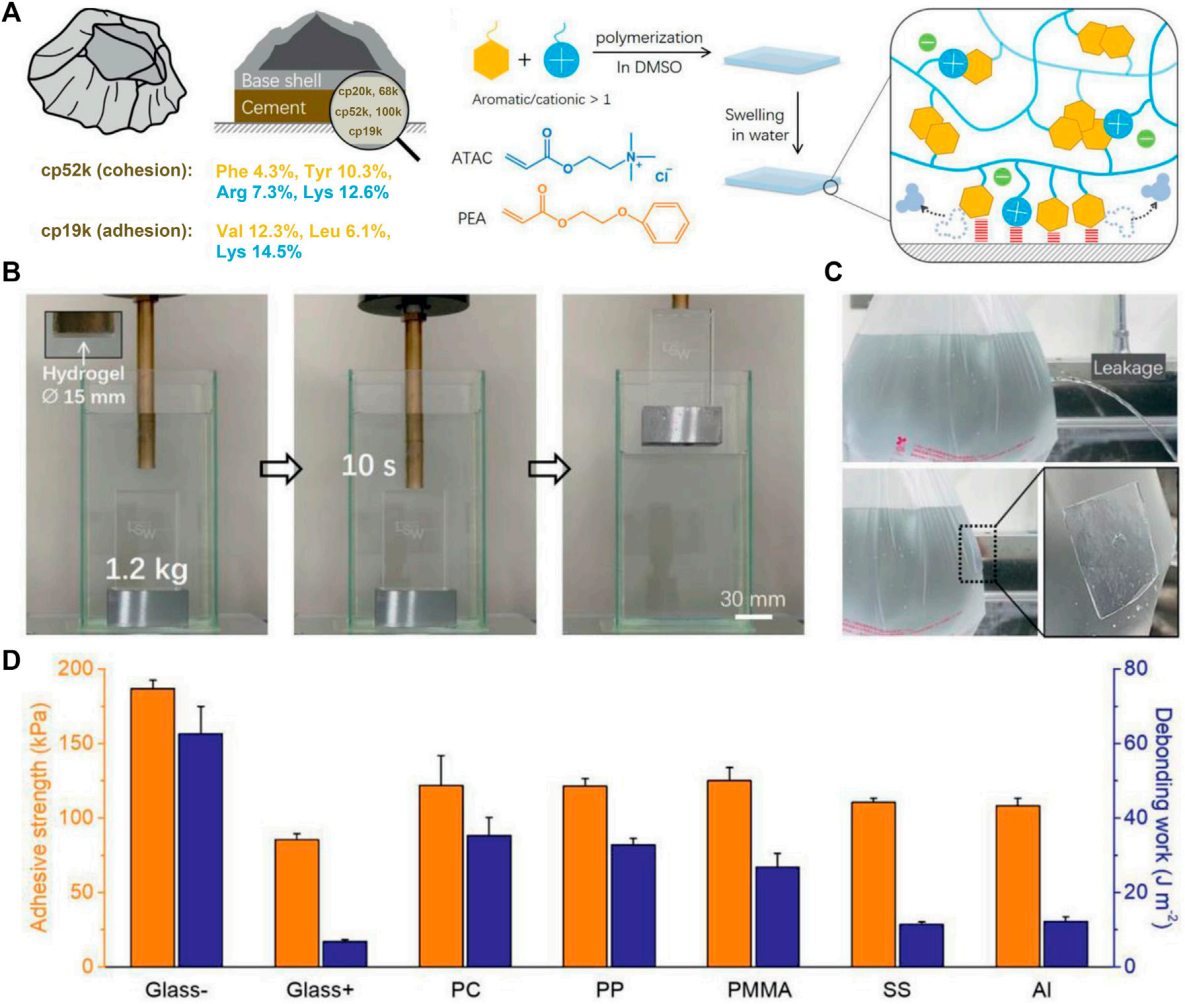
Barnacle cement protein–inspired tough hydrogel showing superior wet adhesion property. **(A)** Schematic representation of the amino acid compositions of cp52k and cp19k and the fabrication process of the hydrogel. **(B)** Photographs of the hydrogel firmly adhering to an underwater object and allowing it to be lifted out of the water. **(C)** Photographs show that the hydrogel immediately repairs a plastic bag of leaking water. **(D)** Adhesion strength of the hydrogel against different substrates in water. Adapted with permission from Fan et al. (2021). Copyright 2021, John Wiley and Sons.

The BCPs located within the bulk cement (e.g., cp52k) have high proportions of cationic Arg and Lys and aromatic Phe and Tyr, which can enhance cohesion by forming strong hydrophobic interactions and cation–π interactions, while the BCPs distributed at the cement interface (e.g., cp19k) contain a lot of cationic Lys and hydrophobic amino acids, which can cooperatively enhance electrostatic attraction–induced adhesion. Inspired by this, Fan et al. (2021) recently fabricated an adhesive hydrogel using cationic 2-(acryloyloxy)ethyl trimethylammonium chloride (ATAC) and aromatic 2-phenoxyethyl acrylate (PEA) as monomers **(** Figure 5A). The hydrogel was obtained by a simple free-radical copolymerization in dimethyl sulfoxide (DMSO), followed by swelling in water. It showed high toughness owing to interchain π−π and cation−π interactions and exhibited excellent wet adhesion capacity attributable to the collaborative adhesion mechanism of interfacial cations and aromatic rings (Maier et al., 2015). The hydrogel could hold onto an underwater object as it was lifted out of the water (Figure 5B) and directly repair a small hole on a plastic bag filled with water to immediately prevent water leakage (Figure 5C **)**. Moreover, it showed strong adhesion on a wide range of different materials **(** Figure 5D). Even after multiple cycles of adhesion and de-adhesion and long periods of immersion, it still maintained its high adhesion strength. To sum up, this hydrogel possesses robust, repeatable, and long-lasting wet adhesion ability, which makes it promising in the fields of rapid underwater repair, underwater object transfer, and wound dressing.

#### 3.3.2 BCIAMs Based on Dopa-Conjugated Amyloid Structures

Since the discovery that barnacles rely on amyloid fibers self-assembled from adhesive proteins for underwater adhesion, researchers have set out to take advantage of amyloid self-assembly to engineer biomimetic adhesive materials. By imitating the non-covalently self-assembling ability of barnacle cement, Nishida et al. (2015) first synthesized a polyacrylamide copolymer adhesive. Their synthesized polymer contains pendant hydroxyl and hexyl groups, which account for interfacial adhesion and self-assembling tetra-alanine units that are responsible for bulk cohesion. By reaction with hexylamine to introduce additional hydrophobic groups at the end of the tetra-alanine unit to activate self-assembly, the polymer solution successfully transformed into a gel within 4 h and showed a maximum dry adhesion strength of approximately 0.4 MPa on poly (methyl methacrylate) plates. The adhesion strength could be further enhanced by optimizing the structure of the polymer.

**FIGURE 6:**
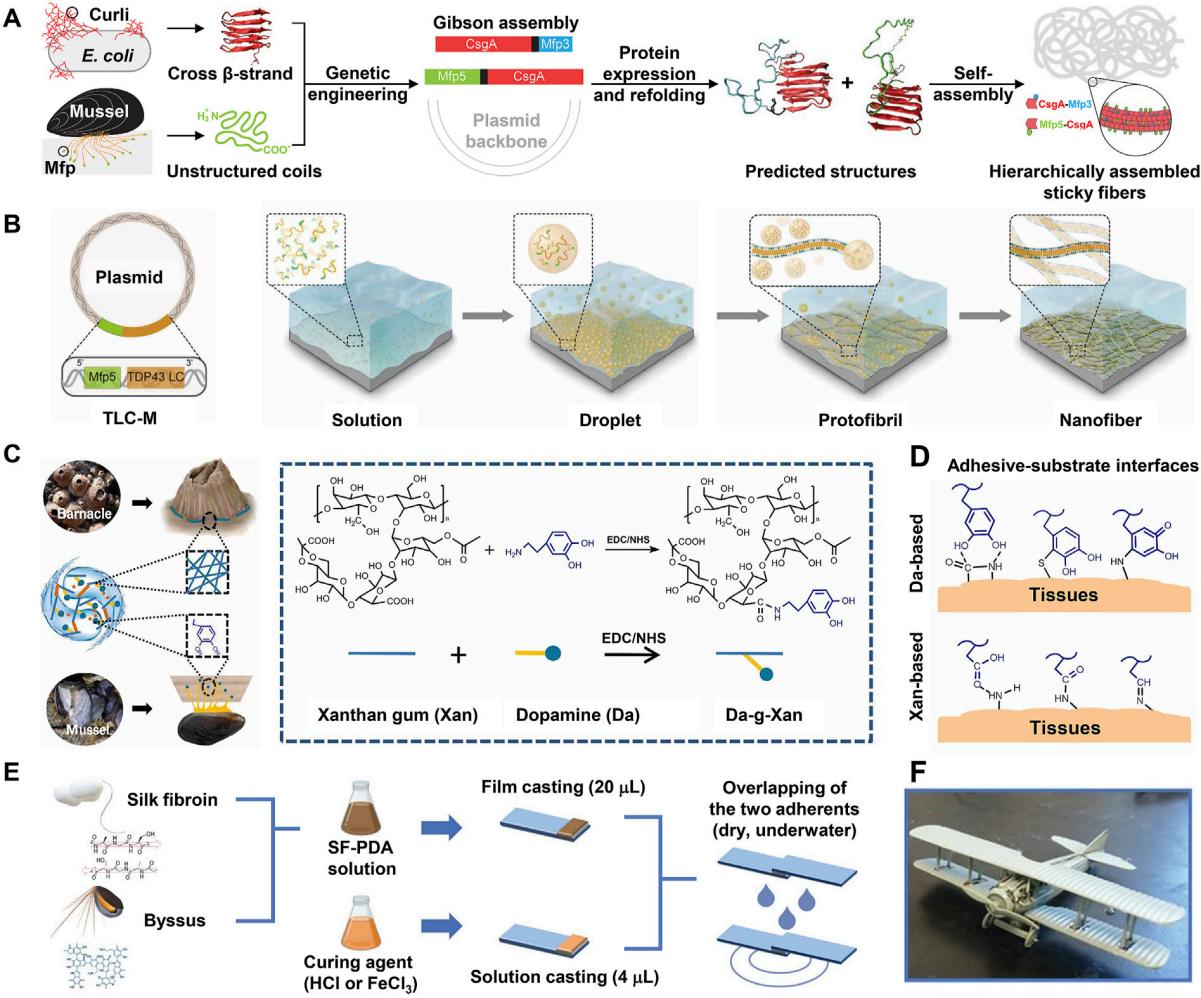
Engineering of BCIAMs based on Dopa-conjugated amyloid structures. **(A)** Combining csgA of bacteria with Mfp3 and Mfp5 of mussels to engineer multi-protein self-assembled sticky nanofibers. **(B)** Engineering of protein-based underwater adhesive materials by pairing a low-complexity (LC) domain of a mammalian DNA-binding protein (TDP43) with Mfp5. Exploiting the LC domain, the protein monomers in the solution first undergo LLPS to form condensed droplets, followed by maturation into nanofibers, and eventually form a stable adhesive layer on the substrate. This mat of nanofibers shows strong underwater adhesion owing to the large surface area and adhesion property of Mfp5 domains. **(C)** Design and synthesis of the Da-g-Xan hydrogel adhesive. EDC: 1-ethyl-3-(3-dimethylaminopropyl)-carbodiimide, NHS: N-hydroxysuccinimide. **(D)** Multiple interfacial linkages between the Da-g-Xan adhesive and tissue surface synergistically mediate strong tissue adhesion. **(E)** Design and lap shearing tests of silk fibroin–polydopamine (SF-PDA) glue. **(F)** Aircraft model assembled by SF-PDA adhesive. **(A)** Adapted with permission from Zhong et al. (2014). Copyright 2014, Nature Publishing Group. **(B)** Adapted from Cui et al. (2019). **(C,D)** Adapted from Huang et al. (2021). **(E,F)** Adapted from Lo Presti et al. (2021).

As the primary role of self-assembly is believed to promote molecular aggregation and enhance cohesion rather than engage in interface adhesion directly, most studies have combined amyloid self-assembly with other adhesion mechanisms (e.g., Dopa surface chemistry) to design biomimetic adhesive materials. A pioneering study (Zhong et al., 2014) in this area engineered a protein-based adhesive that effectively integrates amyloid self-assembly of bacteria and Dopa surface chemistry of mussels **(** Figure 6A). In this study, two fusion proteins (csgA-Mfp3 and Mfp5-csgA) were constructed using the *E. coli* curli protein csgA and mussel foot protein Mfp3 and Mfp5 modules by genetic engineering. In both fusion proteins, the mussel foot proteins adopted disordered conformations beneficial to surface adhesion and they did not disrupt the β-sheet secondary structure of csgA. Accordingly, upon mixing, the two proteins co-assembled into amyloid nanofibers, wherein the csgA formed the backbone and the mussel foot proteins decorated the interface of the nanofibers. This self-assembled multi-protein fibrous adhesive showed excellent underwater adhesion properties with maximum underwater adhesion energy reaching 20.9 mJ/m^2^ as measured by a surface force apparatus, which outperforms any natural mussel foot proteins. Moreover, the fibrous adhesive showed stable adhesion under broad conditions, including alkaline buffers that are known to inhibit Dopa adhesion. Therefore, compared with natural mussel foot proteins, this adhesive made by multi-protein self-assembled nanofibers has much broader applications. Similarly, Cui et al. (2019) designed a fusion protein (TLC-M) by pairing a low-complexity (LC) domain of the mammalian DNA binding protein TDP43 with Mfp5 (Figure 6B). The LC domain has both spontaneous LLPS property and self-assembly ability, which enables the fusion protein monomers in the solution to first undergo LLPS to form condensed liquid-like droplets. Owing to the low surface energy and high protein density, these droplets then readily spread over the surface and rapidly self-assemble into protofibrils, which further mature into dense nanofibers adhering to the substrate (Figure 6B **)**. Exploiting Dopa residues at the exterior of the amyloid core to improve surface adhesion and intermolecular cross-linking, the fusion proteins finally formed a homogenous, dense, and stable adhesive layer on the substrate, whose highest adhesion energy went up to 48.1 mJ/m^2^. Owing to its strong surface wetting and adhesion abilities, the adhesive can be applied for surface modifications of microfluidic channels and underwater repair.

In the aforementioned designs, Dopa residues were converted from Tyr by *in vitro* tyrosinase treatment. Apart from this approach, Dopa functionality can also be obtained by the genetic code expansion technology. This technology is a fast-growing technology in synthetic biology, with which non-canonical amino acids (e.g., Dopa) can be directly incorporated into proteins during their expression *in vivo* (Connor and Tirrell, 2007; Larregola et al., 2012; Yang et al., 2014; Agostini et al., 2017; Budisa and Schneider, 2019). Recently, this technology has also been successfully applied to produce BCIAMs based on Dopa-conjugated amyloid structures. For example, by introducing Dopa into suckersins (squid sucker ring teeth proteins with high β-sheet content) using the genetic code expansion technology, Deepankumar et al. (2020) produced a protein-based adhesive material that combines the amyloid- and Dopa-based adhesive mechanisms. The authors discovered that without Dopa, suckerins alone exhibit outstanding wet adhesion energy (∼15 mJ/m^2^) on mica. In comparison, Dopa-containing suckerins showed more rapid wet adhesion, but the adhesion energy did not show significant enhancement. Kim et al. (2021) engineered a hybrid protein by integrating the self-assembling domain from Aβ, the flexible domain from spider silk proteins, and the adhesive domain (Mfp5) from mussels. They found that under optimized conditions, the protein solution spontaneously undergoes a sol–gel transition to generate a hydrogel triggered by the non-covalent self-assembling process. The obtained hydrogel combines high toughness (approximately 300% extensibility) with strong wet adhesion ability (the maximum underwater adhesion strength on glass substrates reaches 1 MPa) and has potential biocompatibility. Owing to these properties, the authors suggested that this adhesive material is well suited for the repair of tendon-to-bone injuries. Taking advantage of the genetic code expansion technology can not only high efficiently introduce Dopa functionality but also engineer UV-responsive smart adhesives by incorporating photocaged Dopa derivatives (Hauf et al., 2017; Schneider et al., 2017; Schneider et al., 2018).

Chemically conjugating Dopa to polymers with amyloid-like structures is another way to design combinational biomimetic adhesive materials. For instance, using EDC/NHS chemistry, Huang et al. (2021) conjugated Dopa to xanthan gum, an FDA-approved bacterial extracellular polysaccharide that has an intrinsic helical structure mimicking the amyloid fibers of barnacle cement **(** Figure 6C). The resulting Dopa-modified xanthan gum (Da-g-Xan) combined the different adhesion principles of mussels and barnacles and formed an injectable and self-healing hydrogel through abundant intermolecular hydrogen bonds. This hydrogel could achieve multiple linkages with tissues through Dopa- and xanthan gum–based adhesive interactions (Figure 6D). Consequently, it showed higher adhesion strength than commercial fibrin glue on porcine skin. Moreover, its adhesion can be further improved by optimizing the grafting density of Dopa and the total concentration of Da-g-Xan. In rat models, this hydrogel could continuously release Da-g-Xan monomers *in situ* to promote the healing of damaged visceral organs. Therefore, it showed great potential in enhancing recovery following surgical anastomosis. In addition, *via* enzymatic modification or chemical conjugation, a few studies have introduced Dopa to silk fibroin, a protein with rich β-sheet secondary structures, to engineer biomimetic adhesive materials (Burke et al., 2016; Sogawa et al., 2019). As expected, all Dopa-modified silk fibroins demonstrate higher adhesion strength than neat fibroins.

Apart from strong adhesion, a superior adhesive material should also have readily available raw materials, simple fabrication procedures, and environmental friendliness. To this end, Lo Presti et al. (2021) developed a bioinspired adhesive by physically mixing solutions of silk fibroin (SF) and dopamine, which mimic BCPs and mussel foot proteins, respectively (Figure 6E **)**. After aging, dopamine monomers in the mixture were oxidized and polymerized into polydopamine (PDA). The adhesion of polydopamine and silk fibroin alone was weak, whereas their mixture (SF-PDA) showed remarkably increased adhesion owing to the synergy between polydopamine and silk fibroin. In particular, under a high concentration of polydopamine and adding 30 mM FeCl_3_ as a curing agent, SF-PDA firmly adheres to glass slides with a maximum adhesion strength of ∼2.5 MPa in air and ∼2.0 MPa in water, surpassing most biomimetic adhesives. As a proof-of-concept, the different parts were successfully assembled into an intact aircraft model with this adhesive (Figure 6F). This biomimetic adhesive can be easily fabricated with abundant and cheap raw materials, shows high adhesion strength, and is environmentally friendly; as such, it shows great application potential. Likewise, researchers have also fabricated new adhesive materials by simply blending catechol-rich tannic acid with silk fibroin (Bai et al., 2019; Gao et al., 2020; Ke et al., 2020). For example, Gao et al. (2020) developed an adhesive material called TASK composed of tannic acid and silk fibroin. To fabricate TASK, tannic acid and silk fibroin solutions were fully mixed and then freeze-dried, followed by adding water to the lyophilized powder. TASK has good biocompatibility and can adhere to different rabbit tissues and seal a 5 mm incision within minutes, suggesting that it may be a potential medical adhesive.

Functional amyloids (e.g., barnacle cement and bacterial curli fibers) are common in nature. They show good biocompatibility, strong mechanical properties, and interface stability (Knowles and Buehler, 2011; Li et al., 2018). Therefore, intensive research has been carried out to engineer amyloid structure–based biomimetic adhesive materials *via* different approaches. In particular, combining amyloid structures with other adhesion strategies to design BCIAMs has become a major and promising research direction.

#### 3.3.3 BCIAMs Based on the Spatiotemporally Regulated Adhesion Process

**FIGURE 7:**
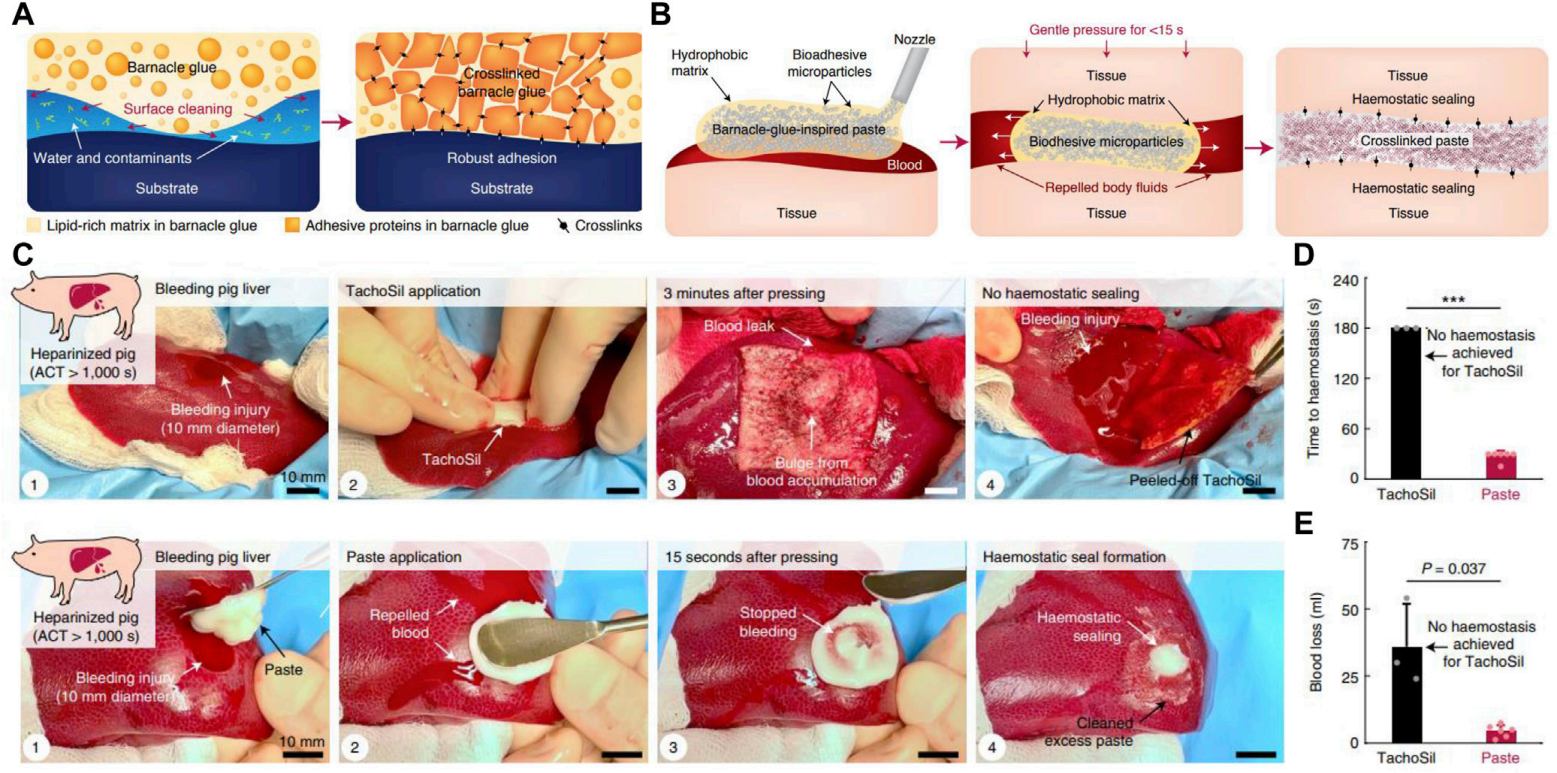
Barnacle cement–inspired paste tissue adhesive. **(A)** Diagram of the adhesion process of barnacle cement. **(B)** Schematic diagram of the application and adhesion mechanism of the barnacle-inspired paste tissue adhesive. **(C)** Paste tissue adhesive successfully prevents bleeding in the injured liver of a pig that was fully anticoagulated after systemic heparin administration [by intravenous heparin bolus to yield an activated clotting time (ACT) of more than 1,000 s], while the commercial product TachoSil failed. **(D)** Time required for TachoSil and paste to stop hemorrhage. **(E)** Final blood loss in hemostasis experiments performed with TachoSil and paste. Adapted from Yuk et al. (2021).

The interfacial hydration layer is considered to be a major obstacle to wet adhesion. To overcome this challenge, barnacles and their larvae first release hydrophobic lipids to remove organic contaminants and water layers on the substrate to create a favorable microenvironment; then, they deposit proteinaceous adhesives to establish robust surface adhesion and intermolecular crosslinks **(** Figure 7A). Inspired by this spatiotemporally regulated adhesion process, Yuk et al. (2021) recently created an injectable paste composed of hydrophobic oils and adhesive microparticles. These adhesive microparticles consisted of crosslinked networks of poly (acrylic acid) grafted with N-hydroxysuccinimide ester (PAA-NHS eater) and chitosan, and had an average diameter of approximately 30 μm. When applied to various bleeding tissues, the barnacle-inspired paste first extruded the oil to repel blood and body fluids under mild (10 kPa) and short (within 15 s) pressure. In the meantime, the adhesive microparticles contacted with each other and the tissue surface, enabling rapid tissue sealing *via* hydrogen bonding. With time, the NHS groups of PAA-NHS ester further reacted with the amine groups of the tissue surface and chitosan to form covalent crosslinks, resulting in more stable and durable tissue sealing (Figure 7B **)**. The paste showed good biocompatibility and strong adhesion ability on diverse wet tissues. *In vivo* experiments further demonstrated that the barnacle-inspired paste could repair a bleeding liver injury and form hemostatic sealing in an anticoagulated pig, with better performance than the commercial hemostatic product TachoSil **(** Figures 7C–E **)**. More importantly, wounds that failed to seal by TachoSil could be quickly repaired with the paste. After surgery with this paste, all pigs survived without obvious signs of adverse reactions. Therefore, it is a promising hemostatic tissue adhesive for clinic applications.

The hydrophobic microenvironment created during barnacle underwater attachment greatly enhances noncovalent interactions between barnacle cement and the substrate, which is one of the reasons why barnacle cement possesses exceptional wet adhesion on a diversity of substrates. Based on this, Li et al. (2020) invented a rapid and simple method to bind hydrogels to diverse polymer substrates. Briefly, this method sandwiched an adhesive layer consisting of 2-ethylhexyl acrylate, isobornyl methacrylate, and SiO_2_ between the hydrogel pregel solution and the substrate. The adhesive layer created a hydrophobic interface to enhance the non-covalent interactions with the substrate and could copolymerize with hydrogel monomers to form an interpenetrating network, thereby firmly connecting the hydrogel to multiple hydrophobic substrates. This method is helpful for expanding the application range of hydrogels.

To summarize, by drawing inspiration from the adhesive principles of barnacle cement, researchers have developed many different BCIAMs *via* different approaches. Their potential applications in technical and biomedical areas have also been explored. However, due to the limited inspirations resulting from the incomplete understanding of barnacle underwater adhesion mechanisms, BCIAMs are much fewer than mussel- and sandcastle worm–inspired adhesive materials. Moreover, most of the designed adhesive materials are proof-of-concept products, and in the future, their design can be further optimized by tuning the polymer backbones, molecular weights, conformations, and so on.

## 4 Summary and Outlook

Barnacle cement relies on unique underwater adhesion strategies that have not been found in mussel foot proteins and sandcastle worm cement proteins to achieve strong surface attachment, which offers new alternatives to inspire engineering new biomimetic adhesive materials that address the challenges of wet adhesion. Accordingly, in recent years, intensive research has been performed to design and develop BCIAMs. However, studies of BCIAMs are much fewer than those of other marine adhesive–inspired adhesive materials primarily because our understanding of barnacle cement underwater adhesion mechanisms remains incomplete, leading to a lack of inspiration. Nevertheless, pioneering studies have successfully engineered different categories of BCIAMs, including biomimetic surface anchors based on natural barnacle cement, recombinant BCPs as well as barnacle-inspired peptides designed in a reductionist way, and biomimetic adhesive materials inspired by the adhesive principles of barnacle cement. Throughout these studies, numerous adhesive materials have been developed, but they are far from satisfactory. Therefore, the underwater adhesion mechanisms of barnacle cement and the optimized design and fabrication of BCIAMs should be focused on in future studies.

By summarizing the research progress of BCIAMs, several promising research directions have been identified. They include (1) design, synthesis, and application of barnacle-inspired self-assembling peptides. Most BCPs have the special capacity to self-assemble into amyloid fibers, differentiating them from mussel and sandcastle worm adhesive proteins. Therefore, design and self-assembly studies of barnacle-inspired peptides can not only advance our knowledge on the structure and functional relationships of different BCPs in a reductionist way but also expand peptide self-assembly research by discovering new self-assembling peptide units. (2) Research on amyloid structure–based biomimetic adhesive materials. Wet adhesion *via* Dopa-dependent or Dopa-independent amyloid structure–based glues are two important pathways in nature. Fabricating Dopa-dependent adhesive materials has encountered several challenges, such as complicated synthesis procedures and loss of adhesion due to the oxidation of Dopa (Ahn, 2017). In contrast, amyloid structure–based adhesive materials can be readily made on a large scale from commercially available materials; therefore, they have outstanding application potential. However, this research is still in its infancy, and more efforts are needed to clarify the adhesion mechanism and explore different ways to fabricate biomimetic adhesive materials. (3) Engineering of new bioinspired adhesive materials by integrating multiple adhesion strategies. Integrating diversified underwater adhesion mechanisms such as LLPS, Dopa chemistry, amyloid structure, and organic–inorganic hybridization, to engineer multifunctional biomimetic adhesive materials, is critical for creating artificial adhesives that have better adhesion than their natural counterparts. The study of Cui et al. (2019) has witnessed the miraculous power of this approach. In the future, the design and fabrication of multifunctional biomimetic adhesive materials should be optimized to obtain simple fabrication procedures while maintaining strong adhesion. The modular design and genetic code expansion technology of synthetic biology offer powerful tools toward this goal. (4) Biomimicry of biological regulation of the barnacle adhesion process. Increasing evidence has shown that biological adhesives not only depend on their special biochemical compositions and microscale structures but also on the spatiotemporal regulation during the adhesion process. Unfortunately, most studies design biomimetic adhesives only by learning from the compositions and structures of bioadhesives, leading to relatively weak adhesion of biomimetic adhesive materials. Recently, Yuk et al. (2021) engineered a paste with better tissue adhesion than commercial hemostatic agents in the sealing of bleeding porcine, inspired by the regulated adhesion process of barnacles. Their results indicate that imitating the spatiotemporal regulation measures of bioadhesives in the design of biomimetic adhesive materials offers great potential.
